# Fibroblast-Targeted Nanodelivery Systems: Mechanisms of Collagen Remodeling Regulation and Novel Strategies for Scar Repair

**DOI:** 10.3390/pharmaceutics18020172

**Published:** 2026-01-28

**Authors:** Junshan Lan, Zhipeng Teng, Qian Huang, Fang Qin, Yibin Zheng, Yuting Liu, Yilin Chang, Xing Zhou, Xiaohui Li, Wenwu Wan, Lu Wang, Jie Lou

**Affiliations:** 1 School of Pharmacy and Bioengineering, Chongqing University of Technology, Chongqing 400054, China; 17783568946@163.com (J.L.); 15306896995@163.com (Q.H.); qf202419@163.com (F.Q.); zhengyobi@163.com (Y.Z.); liuyuting0404@163.com (Y.L.); 19923250735@139.com (Y.C.); 2 Department of Neurosurgery, Chongqing Hospital of Traditional Chinese Medicine, Chongqing 400011, China; tengzhipeng@cdutcm.edu.cn (Z.T.); wanwew520@163.com (W.W.); 3 Yunnan Key Laboratory of Stem Cell and Regenerative Medicine, School of Rehabilitation, Kunming Medical University, Kunming 650500, China; zhouxing@kmmu.edu.cn; 4 Department of Pharmaceutics, College of Pharmacy, Army Medical University, Chongqing 400038, China; lpsh008@aliyun.com

**Keywords:** fibroblasts, scar formation, signal regulation, nanodrug delivery systems

## Abstract

Scar formation is a common outcome of post-injury repair and can compromise both esthetic appearance and physiological function. Fibroblasts are central mediators of this process; their aberrant activation or differentiation into myofibroblasts drives fibrosis and excessive scar tissue accumulation. Nanodrug delivery systems (NDDSs) offer unique opportunities to modulate fibroblast behavior through cell-/microenvironment-guided targeting, controlled release, and stimuli-adaptive designs. Here, we summarize fibroblast biology across scar repair and delineate the mechanistic underpinnings of scar pathogenesis. We then synthesize recent progress in NDDS-enabled interventions for pathological scarring, with an emphasis on how materials design can be matched to fibroblast states and wound-stage cues. By connecting mechanisms to delivery strategies, this review provides a framework to guide the development of scar-minimizing therapies and functional tissue regeneration.

## 1. Introduction

Scar formation represents the body’s reparative response to tissue injury, characterized by aberrant collagen deposition and dysregulated extracellular matrix (ECM) remodeling [1]. Although scarring is integral to wound closure, the resulting tissue often lacks the structural integrity and functional properties of native skin, leading to esthetic and physiological impairments [2]. In severe cases, these deficits impose substantial psychosocial and functional burdens, highlighting the urgent clinical need for effective anti-scarring strategies. A central challenge in regenerative medicine is thus to promote functional tissue regeneration while simultaneously preventing pathological scar formation.

Fibroblasts are the principal effector cells in wound healing, regulating inflammation, ECM synthesis and degradation, angiogenesis, and tissue remodeling [3,4]. Upon activation and differentiation into myofibroblasts, fibroblasts acquire contractile properties that facilitate wound contraction but also reshape the tissue mechanical microenvironment [5]. Following injury, these cells migrate, proliferate, and produce large quantities of ECM components, particularly collagens. However, excessive or sustained activation leads to an imbalance between collagen synthesis and degradation, resulting in the accumulation of disorganized fibers that underlie hypertrophic scars (HS) and keloids [6]. Therefore, precise modulation of fibroblast activation, phenotype, and matrix remodeling dynamics constitutes a rational therapeutic avenue to improve scar outcomes.

Despite advances in topical formulations, injectable agents, and surgical techniques, current anti-scar therapies remain constrained by three key barriers. First, poor transdermal penetration restricts the delivery of most agents to the dermis, where activated fibroblasts reside. Second, inadequate targeting results in suboptimal drug accumulation at the site of action and limited engagement of pathological fibroblasts. Third, imprecise release profiles can lead to insufficient local exposure during critical therapeutic windows. Collectively, these limitations motivate more sophisticated delivery strategies.

In recent years, nanodrug delivery systems (NDDSs), such as liposomes, polymeric nanoparticles, micelles, and inorganic nanocarriers, have emerged as promising platforms to overcome these barriers. Surface engineering with penetration enhancers and ligand-directed motifs can improve transdermal transport and achieve selective binding to activated fibroblasts, thereby enhancing local bioavailability [7]. Stimuli-responsive architectures enable on-demand drug release in response to wound-specific cues such as pH changes, elevated ROS, enzymatic activity, and altered mechanical properties, thereby aligning therapeutic action with the spatiotemporal progression of wound healing [8,9,10]. NDDSs also protect labile cargos from degradation and can prolong in vivo half-life, improving exposure at the target site [11].

This review critically examines the central role of fibroblasts in scar pathogenesis and synthesizes recent progress in NDDSs for scar management. By linking materials design to fibroblast-centered mechanisms and measurable outcomes, this review aims to provide a coherent framework for advancing nanotechnology-driven anti-scar interventions from concept to clinic.

Importantly, this review explicitly integrates emerging insights into fibroblast heterogeneity and phenotypic plasticity into the discussion of NDDSs, highlighting how ligand-mediated targeting, stimuli-responsive release, and microenvironment-sensing strategies can be leveraged to selectively modulate pathological fibroblast subpopulations while preserving normal reparative fibroblast function during wound healing and scar maturation.

## 2. Wound Healing and Scarring

**Figure 1:**
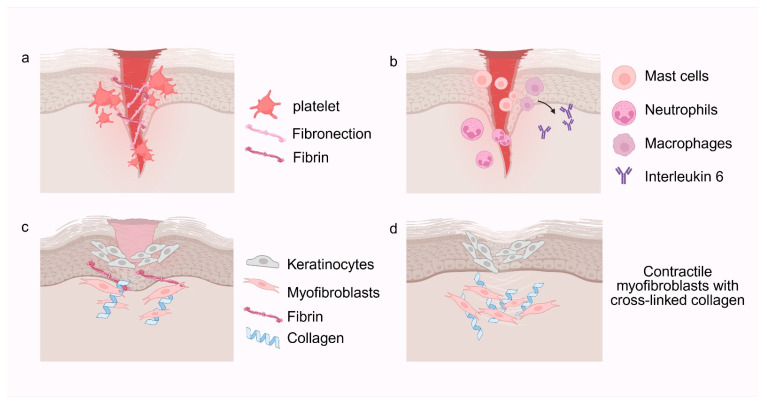
Stages of wound healing. (**a**) Hemostasis: Platelets aggregate at the wound site, forming a clot and releasing growth factors. Platelets also recruit inflammatory cells such as neutrophils and macrophages. (**b**) Inflammation: Neutrophils clear pathogens and debris, whereas macrophages coordinate inflammation resolution and activate fibroblasts. Mast cells release cytokines that amplify inflammation. (**c**) Proliferation: Keratinocytes migrate and proliferate to re-epithelialize the wound. Fibroblasts secrete collagen and other ECM components, and a subset differentiates into myofibroblasts to drive wound contraction. (**d**) Remodeling: Fibroblasts and myofibroblasts remodel the ECM by realigning collagen fibers and restoring matrix homeostasis, leading to mature scar formation. Created in BioRender. Huang, Q. (2026) https://BioRender.com/4lwgeuy.

Scar formation is a multistage and dynamically regulated process involving coordinated physiological and biochemical events during tissue repair [12]. Figure 1 provides an overview of the canonical phases of wound healing and their key cellular events. Following cutaneous injury, a tightly orchestrated cascade of repair mechanisms is rapidly activated to restore tissue integrity and function. However, dysregulation of these reparative responses can shift healing toward aberrant fibrotic proliferation and excessive scar deposition. Accumulating evidence indicates that scar development arises from the convergence of multiple factors, including prolonged wound duration, persistent inflammation, excessive collagen deposition, and impaired ECM remodeling [13].

The inflammatory phase is initiated immediately following injury and generally persists for 3 to 5 days [3]. Initially, platelets aggregate at the injury site, releasing coagulation factors and forming a fibrin network that generates a hemostatic clot. Beyond initiating hemostasis, platelets release a spectrum of cytokines and growth factors, including transforming growth factor-β (TGF-β) and platelet-derived growth factor (PDGF), which recruit inflammatory cells to the wound site [14]. Subsequently, immune cells such as neutrophils and macrophages are recruited to the injured area. Neutrophils act to eradicate pathogens and clear cellular debris, whereas macrophages, after completing phagocytic clearance, secrete growth factors that stimulate fibroblast migration, proliferation, and activation. These events establish a pro-regenerative yet inflammation-dominated microenvironment that primes subsequent cellular proliferation and tissue remodeling.

The proliferative phase typically spans 3 to 4 weeks. During this stage, fibroblasts undergo activation and direct migration into the wound bed, where they initiate robust synthesis of collagen and other ECM components [15]. Fibroblasts serve as key effector cells during this phase by contributing to the formation of granulation tissue, a vascularized connective matrix that is essential for tissue regeneration [16]. Angiogenesis plays a vital role in ensuring adequate oxygen and nutrient delivery to the regenerating tissue. As epithelial cells migrate from the wound edges to cover the granulation tissue, epithelialization restores skin barrier integrity. Simultaneously, myofibroblasts mediate wound contraction, thereby facilitating wound closure. However, excessive or sustained myofibroblast-mediated contraction can distort tissue architecture and compromise functional recovery, particularly in mechanically active regions.

The final stage, scar maturation, involves the transformation of granulation tissue into mature scar tissue. Collagen fibers undergo substantial remodeling, with type III collagen being gradually replaced by mechanically stronger type I collagen. Although elastin, a key protein responsible for skin elasticity, is partially restored during this phase, its regeneration remains incomplete in deep wounds, thereby contributing to structural abnormalities. Programmed apoptosis eliminates excessive vascular cells and myofibroblasts, thereby terminating active repair and consolidating scar architecture.

## 3. The Role of Fibroblasts in Scar Formation

Fibroblasts act as central regulators of tissue repair and scar pathogenesis by integrating inflammatory, biochemical, and mechanical cues. They influence scar development and phenotype through mechanisms including cytokine production, modulation of ECM homeostasis, and differentiation into myofibroblasts.

### 3.1. Biological Functions of Fibroblast

Fibroblasts constitute the predominant stromal cell population within connective tissues and serve as key orchestrators of tissue homeostasis and repair [17]. Marked heterogeneity exists among fibroblasts across developmental stages and anatomical sites, giving rise to specialized subpopulations that support skin development, homeostasis, and wound repair [18,19]. Single-cell RNA sequencing further delineates organ-specific states—including cardiac, hepatic, and dermal fibroblasts, as well as derivatives from induced pluripotent stem cells (iPSCs) [20]. This phenotypic and functional diversity reflects tissue context, regional cues, microenvironmental signals, and dynamic cell states [21].

Across organs, fibroblasts contribute to both physiological repair and pathological fibrosis. In the heart, injury induces differentiation into myofibroblasts that secrete ECM, influencing scar formation and cardiac performance [22]. In the liver, fibroblasts participate in fibrogenic signaling networks that drive disease progression [23]. In skin, fibroblasts coordinate wound healing and scar formation and modulate inflammation through growth-factor and cytokine release [24]. Functionally, they synthesize and remodel ECM components (e.g., collagens, elastin) and regulate cell migration, proliferation, and apoptosis [25]. During repair, fibroblast-derived factors such as basic fibroblast growth factor (bFGF) and TGF-β promote re-epithelialization and granulation [26]. Concurrently, cytokines and chemokines—including interleukin-6 (IL-6), tumor necrosis factor-α (TNF-α), CCL2, and CXCL8—govern immune-cell recruitment and activation [27]. When activation becomes sustained, fibroblasts drive excessive ECM deposition, culminating in HS and keloids [28,29]. A precise understanding of fibroblast biology and its pathological reprogramming is therefore essential for targeted anti-scarring strategies.

Beyond static classification, fibroblast subpopulations undergo dynamic phenotypic switching throughout wound healing and scar maturation, transitioning between quiescent, activated, pro-fibrotic, and remodeling-associated states in response to biochemical and mechanical cues. This temporal plasticity implies that effective therapeutic intervention should not rely on a single, fixed fibroblast marker, but rather exploit microenvironment-dependent features such as redox imbalance, matrix stiffness, enzymatic activity, and cytokine gradients. These characteristics provide a biological rationale for the design of NDDSs capable of stage-adaptive targeting, enabling selective modulation of pathological fibroblasts at specific phases without disrupting physiological repair processes.

### 3.2. Promotion of Cytokine Synthesis and Secretion

**Figure 2:**
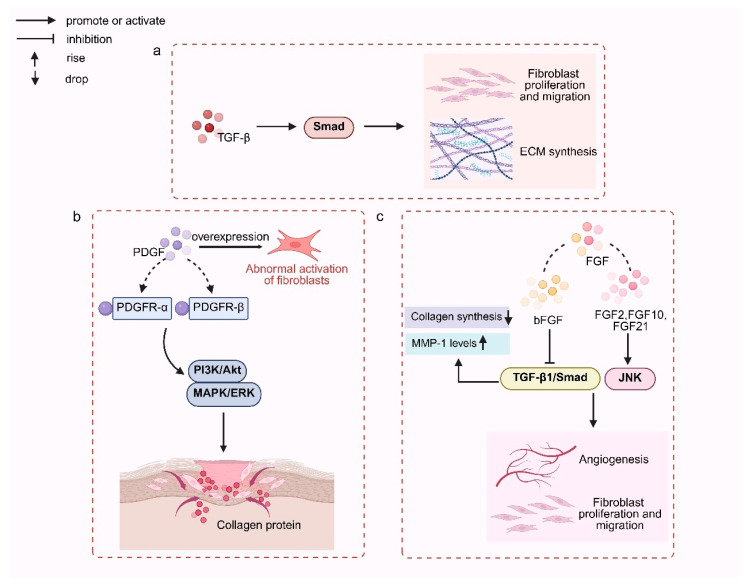
Fibroblasts synthesize and secrete cytokines. (**a**) The TGF-β pathway activates Smad proteins, leading to fibroblast proliferation, migration, and ECM synthesis, contributing to wound healing. (**b**) The overexpression of PDGF induces the abnormal activation of fibroblasts through PDGFR-α and PDGFR-β, which activates downstream PI3K/Akt and MAPK/ERK signaling pathways, promoting collagen protein synthesis and tissue remodeling. (**c**) FGFs, particularly through bFGF and other FGF family members (FGF2, FGF10, FGF21), interacts with TGF-β1/Smad and JNK pathways, regulating angiogenesis, collagen synthesis, and fibroblast activity.Created in BioRender. Huang, Q. (2026) https://BioRender.com/tkv3alm.

Fibroblasts actively reshape the wound microenvironment by synthesizing and secreting a network of cytokines and growth factors, among which TGF-β, PDGF, and fibroblast growth factors (FGFs) play dominant roles in regulating inflammation, cell recruitment, and ECM dynamics. As summarized in Figure 2, fibroblasts actively synthesize and secrete a broad spectrum of cytokines that shape the wound microenvironment during tissue repair.

#### 3.2.1. TGF-β

TGF-β is a core regulatory factor in scar formation. Its canonical Smad signaling pathway activates Smad2/3 phosphorylation, forming heterodimers that translocate to the nucleus to regulate the expression of genes related to fibroblast proliferation, migration, and ECM synthesis. Dysregulation of this pathway drives fibrosis progression. Current therapeutic strategies targeting this pathway include neutralizing antibodies, ligand traps, and receptor kinase inhibitors, aiming to block abnormal signal transduction [30]. Additionally, TGF-β can enhance its own expression through an autocrine loop, forming a profibrotic positive feedback, which is also a key mechanism for the persistent progression of scars.

#### 3.2.2. PDGF

PDGF recruits and expands fibroblasts, smooth muscle cells, and mononuclear cells at sites of injury, thereby supporting connective-tissue growth and scar development. Binding to PDGFR-α/β activates PI3K/Akt and MAPK/ERK pathways that regulate fibroblast proliferation and migration [31]. Experimental studies show that PDGF enhances fibroblast motility and growth, accelerating wound closure [32]. PDGF signaling also augments ECM biosynthesis, particularly collagen, and modulates fibroblast-mediated collagen expression during remodeling. However, PDGF overexpression is linked to aberrant fibroblast activation and pathological scarring [33].

#### 3.2.3. FGFs

FGFs facilitate wound healing by stimulating fibroblast proliferation, migration, and angiogenesis and by improving scar quality through regulated ECM synthesis and remodeling. In skin, FGF2, FGF7, FGF10, and FGF21 are highly expressed, FGF2/10/21 promote fibroblast migration and activate JNK signaling [34]. bFGF exerts antifibrotic effects by inhibiting the aforementioned TGF-β1/Smad axis, reducing collagen synthesis, upregulating Matrix Metalloproteinase-1 (MMP-1) activity, and activating the ERK and Akt signaling pathways to promote fibroblast proliferation and DNA synthesis, thereby improving the structure and pigmentation of scar tissue [35,36]. In vivo experiments have confirmed that bFGF-loaded hydrogels can significantly promote wound repair and regulate collagen remodeling [37].

### 3.3. Regulation of ECM Synthesis and Degradation

**Figure 3:**
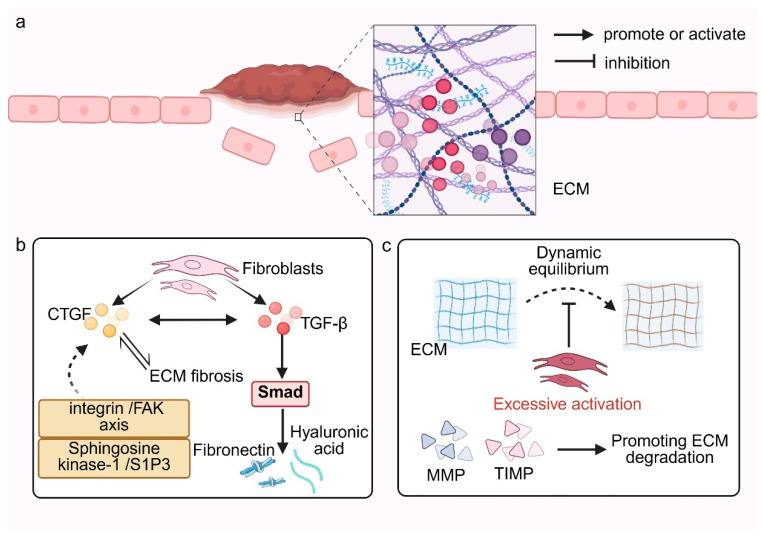
Diagram of the mechanisms by which fibroblasts regulate ECM synthesis and degradation. (**a**) Activated fibroblasts accumulate within the wound ECM, driving excessive matrix deposition and increased tissue stiffness. (**b**) Fibroblasts promote ECM synthesis through TGF-β/Smad and CTGF signaling, with CTGF further enhancing fibrogenesis via the integrin/FAK and SphK1/S1P3 axes. (**c**) Persistent fibroblast overactivation disrupts the MMP/TIMP balance, impairing ECM degradation and leading to progressive fibrosis. Created in BioRender. Huang, Q. (2026) https://BioRender.com/zp9v3yd.

ECM is a highly organized three-dimensional network composed primarily of collagens, elastin, fibronectin (FN), laminins, proteoglycans, glycosaminoglycans, and associated glycoproteins. It provides structural support and governs essential processes including cell adhesion, migration, proliferation, differentiation, and signal transduction [38]. Fibroblasts are the principal cellular source of ECM components. During scar formation, production of type I and III collagens, FN, and elastin is markedly upregulated, resulting in excessive matrix deposition, tissue stiffening, and fibrosis [39,40]. Figure 3 summarizes how activated fibroblasts promote fibrosis by enhancing ECM synthesis while impairing matrix degradation.

This process is modulated by cytokines such as TGF-β and connective tissue growth factor (CTGF/CCN2). TGF-β enhances COL1A1 expression through canonical Smad signaling and stimulates FN and hyaluronan secretion, thereby increasing ECM mechanical strength and stability [41,42,43]. CTGF acts synergistically with TGF-β to further amplify ECM synthesis and promote myofibroblast differentiation, establishing a positive feedback loop that accelerates fibrotic progression [44,45]. Notably, a fibrotic matrix can itself augment CTGF expression, reinforcing this feed-forward cycle. Mechanistically, CTGF engages integrin/focal adhesion kinase (FAK) signaling and the sphingosine kinase-1/S1P3 pathway to potentiate these effects [46].

Under physiological conditions, ECM synthesis and degradation are tightly balanced to preserve tissue architecture, whereas disruption of this equilibrium during wound healing drives pathological matrix accumulation and fibrosis [47]. MMPs and their endogenous inhibitors, tissue inhibitors of metalloproteinases (TIMPs), are central to this balance. Although MMPs are traditionally viewed as antifibrotic owing to their proteolytic activity, context-dependent roles have emerged in which specific MMPs can facilitate fibrosis [48,49]. The mechanical properties of the ECM, together with TGF-β1 signaling, further calibrate fibroblast-mediated remodeling.

Additional regulators modulate this network. Endostatin attenuates fibrosis at least in part by dampening PDGFR/ERK signaling. Therapeutically, tuning the MMP–TIMP axis and the competitive interplay between MMPs and lysyl oxidase (LOX), a key crosslinking enzyme, has been proposed to restore matrix homeostasis and restrain fibrotic progression [50,51].

### 3.4. Regulation of Inflammatory Cytokine Secretion

**Figure 4:**
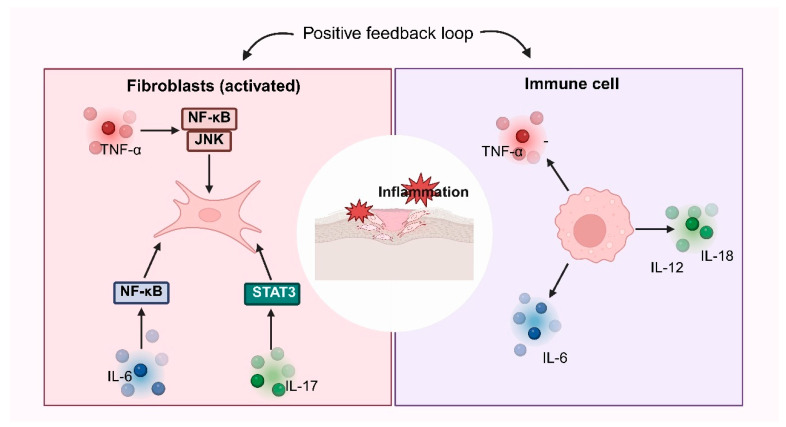
Fibroblasts secrete inflammatory factors. Fibroblasts release TNF-α, IL-6, and IL-1β, activating NF-κB, JNK, and STAT3 pathways to enhance proliferation, ECM synthesis, and inflammation. Immune cells amplify this response through a positive feedback loop, sustaining chronic inflammation and promoting pathological fibrosis. Created in BioRender. Huang, Q. (2026) https://BioRender.com/e3i6uv9.

During scar formation, fibroblasts secrete pro-inflammatory cytokines, including TNF-α, IL-6, and IL-1β, that act through autocrine and paracrine circuits to stimulate neighboring fibroblasts and immune cells, creating a self-reinforcing inflammatory milieu that accelerates fibrosis. A schematic overview of fibroblast-driven inflammatory amplification loops is provided in Figure 4.

#### 3.4.1. TNF-α

TNF-α promotes fibroblast proliferation, migration, and ECM protein synthesis mainly by activating NF-κB, together with stress kinases (JNK and p38 MAPK), thereby amplifying inflammatory signaling [52]. Rather than reiterating the full NF-κB cascade, it is important to emphasize its scar-relevant consequence: persistent NF-κB activity sustains cytokine transcription, cooperates with profibrotic programs, and is associated with increased collagen production and aberrant healing trajectories [53,54].

#### 3.4.2. IL-1 Family and Pathway Crosstalk

Interactions between NF-κB, TGF-β, and IL-1 signaling are integral to fibroblast activation. IL-1 enhances fibroblast proliferation predominantly through NF-κB, with chronic wounds exhibiting heightened NF-κB–IL-1 interplay that amplifies inflammation and impairs tissue regeneration [55,56]. This signaling complexity illustrates how multiple cytokines converge to direct fibroblast behavior and repair outcomes.

#### 3.4.3. Additional Cytokines

IL-1β augments fibroblast proliferation, ECM production, and inflammatory cell recruitment, thereby reinforcing a profibrotic microenvironment [57]. Type 2 cytokines, including IL-4 and IL-13, promote fibrosis by upregulating periostin expression in dermal fibroblasts, which in turn induces TGF-β1 via RhoA/ROCK signaling [58]. IL-17 enhances expression of stromal cell-derived factor-1 (SDF-1/CXCL12) and other profibrotic mediators in keloid fibroblasts through STAT3 activation [59]. These axes exemplify the broader immune–stromal dialog that sustains fibrosis. The inflammatory microenvironment, characterized by infiltration of macrophages, mast cells, and T lymphocytes, supports the view of keloids as an inflammatory skin disorder [60]. Elucidating these networks provides a rationale for cytokine-targeted immunotherapies to mitigate inflammation and limit hypertrophic and keloid scarring [61].

## 4. Fibroblast-to-Myofibroblast Transition

In uninjured adult skin, fibroblasts remain largely quiescent, exhibiting limited migratory and proliferative activity. Following tissue damage, fibroblasts are recruited to the wound bed and differentiate into contractile myofibroblasts characterized by α-smooth muscle actin (α-SMA) expression. Through coordinated contractile activity and matrix synthesis, myofibroblasts drive wound contraction and tissue repair; however, excessive or persistent activation underlies pathological scarring [62].

### 4.1. Cellular Origins of Myofibroblasts

Current evidence supports three principal sources of myofibroblasts in cutaneous repair: (i) activation of resident interstitial fibroblasts, which exit quiescence under traumatic or inflammatory cues and acquire a myofibroblast phenotype; (ii) EMT, whereby epithelial cells adopt mesenchymal features and contribute to the myofibroblast pool; and (iii) bone marrow-derived progenitors that differentiate systemically and home to the wound via the circulation [63].

### 4.2. Core Signaling Programs

Differentiation of fibroblasts into myofibroblasts constitutes a pivotal event in scar formation, integrating excessive ECM deposition with α-SMA-dependent contractility and sustained tissue tension. As discussed above, this phenotypic transition is primarily governed by TGF-β/Smad signaling and further shaped by mechanical cues and inflammatory cytokine networks [64].

Beyond the core pathway itself, recent studies have identified regulatory nodes that fine-tune TGF-β/Smad-driven myofibroblast activation. Eukaryotic translation initiation factor 6 (eIF6) acts as an upstream suppressor by limiting Smad2/3 phosphorylation, thereby restraining myofibroblast differentiation [65]. In contrast, small ubiquitin-like modifier 1 (SUMO1), which is upregulated in HS, enhances Smad4 SUMOylation and nuclear retention, amplifying profibrotic gene transcription and prolonging myofibroblast activation [66]. Together, these findings highlight that sustained scarring is not solely dictated by TGF-β availability but is critically influenced by pathway-specific modulators that reinforce or constrain fibrotic signaling outputs.

### 4.3. Mechanotransduction and Matrix Cues

Mechanical forces are decisive determinants of fibroblast fate. Tensile stress at the wound edge facilitates myofibroblast differentiation, while resolution of mechanical load during maturation can favor reversion toward a fibroblast state. Pathological scarring correlates with persistent activation of mechanotransduction pathways, notably FAK and the RhoA/ROCK cascade, which enhance contractile function, cytoskeletal integrity, and myofibroblast survival [67,68].

Extracellular tension also activates the transcriptional co-activators YAP and TAZ, which synergistically regulate contractility and pro-fibrotic gene expression; dual inhibition yields stronger antifibrotic effects than single-gene targeting. YAP/TAZ integrate inputs from TGF-β, WNT, and other pathways to coordinate myofibroblast differentiation and function [69]. Beyond skin, they mediate stiffness sensing and stem cell activation in skeletal muscle after injury [70], and modulate ECM homeostasis via “mechanical epigenetics”, wherein YAP overexpression preserves chromatin architecture and suppresses catabolic programs under reduced tension [71]. Together, these findings position YAP/TAZ as central transducers linking physical cues to fibrotic gene regulation.

### 4.4. Adhesion Complexes and Fibronectin–Integrin Signaling

FN and integrins are key effectors of force transmission and signaling during myofibroblast activation. Assembly of FN fibers via integrin α5β1 organizes the integrin–actin axis and supports cellular contractility [72]. Integrin subunits, particularly β1 and αv, sense matrix mechanics and activate intracellular pathways such as FAK/Src that govern migration, survival, and fibrogenic progression [73,74]. FN–integrin engagement further stimulates RhoA/ROCK signaling to reinforce myofibroblast differentiation [75]. The integrin adhesion complex (IAC) functions as a hub that integrates biochemical and mechanical inputs to regulate cytoskeletal dynamics, transcriptional programs, and cell fate [76]. In cardiomyocytes, illustrative of broader principles, adhesion plaques and associated proteins direct mechanotransduction and influence lineage decisions, underscoring the conserved role of adhesion machinery in mechano-regulated differentiation [77].

## 5. Nanodrug Delivery Systems for Scar Intervention

**Figure 5:**
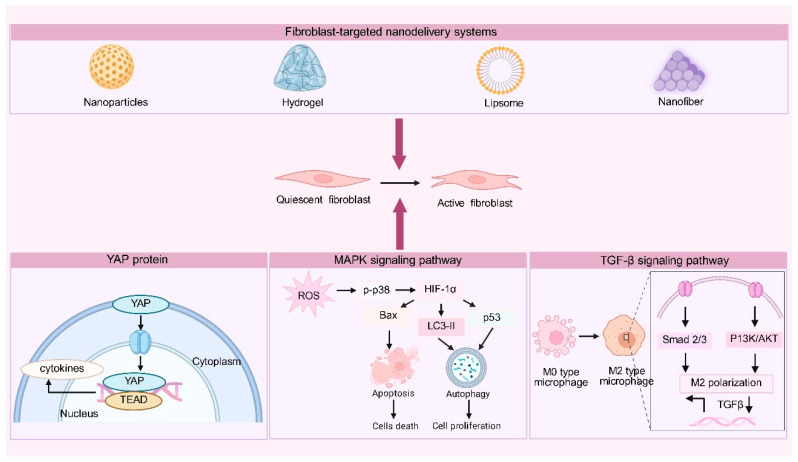
Integrative schematic linking key signaling pathways and intervention points of fibroblast-targeted NDDSs. Representative NDDS platforms, including nanoparticles, hydrogels, liposomes, and nanofibers, enable targeted delivery to fibroblasts and promote the transition from quiescent to activated states. YAP-mediated mechanotransduction regulates fibroblast transcription via YAP–TEAD complex formation, while ROS-activated MAPK signaling controls p38 phosphorylation and HIF-1α-dependent balancing of LC3-II (microtubule-associated protein 1 light chain 3-II)-associated autophagy and Bax-mediated apoptosis, thereby determining fibroblast proliferation, survival, or cell death. In addition, NDDS-mediated immunomodulation promotes M2 macrophage polarization through TGF-β–associated Smad2/3 and PI3K/AKT signaling, indirectly supporting fibroblast activation and tissue repair. Created in BioRender. Huang, Q. (2026) https://BioRender.com/ghlfwlx.

Building on the fibroblast-centered mechanisms discussed above, it is increasingly evident that effective scar modulation requires delivery strategies capable of interfacing with multiple regulatory layers simultaneously, including intracellular signaling, mechanotransduction, and immune–stromal crosstalk. To elucidate how NDDSs participate in the regulation of these complex biological processes, an integrative schematic (Figure 5) is provided, summarizing fibroblast-associated signaling pathways and representative NDDSs. This framework highlights fibroblasts as central therapeutic targets and illustrates how rational NDDS design can be leveraged to modulate fibroblast activation, survival, and function during scar formation and tissue repair.

Current clinical interventions for abnormal cutaneous scarring include pressure dressings, silicone gel sheets, corticosteroid injections, laser therapy, and surgical excision [78]. However, these approaches exhibit limitations such as variable efficacy, high recurrence rates, and notable side effects. While corticosteroid injections can transiently suppress fibroblast proliferation, they often cause adverse effects including skin atrophy, dyspigmentation, and localized infections. Surgical removal may also trigger scar recurrence due to stress-induced tissue regeneration. These limitations underscore the need for strategies that combine efficacy with low toxicity and that actively support functional tissue regeneration.

Advances in nanotechnology have yielded NDDSs with tunable size, surface chemistry, and release profiles, enabling improved negotiation of the skin barrier, selective engagement of pathological fibroblasts, and synchronization of payload availability with wound-healing dynamics [79]. Increasing evidence indicates that NDDSs modulate fibroblast behavior by inducing apoptosis, suppressing migration and proliferation, regulating key signaling pathways such as TGF-β/Smad, and altering inflammatory and mechanically stressed microenvironments [80,81]. Collectively, fibroblast-targeted NDDSs offer a mechanistically grounded route to scar prevention and treatment while advancing the goal of functional, low-scar skin repair.

### 5.1. Nanoparticles

Nanoparticles possess unique physicochemical features, including nanoscale dimensions, high surface-to-volume ratios, and tunable surface chemistry, which together enable enhanced interaction with biological barriers and precise regulation of cellular processes [82]. Through rational engineering, nanoparticle surfaces can be functionalized with targeting ligands, penetration enhancers, or responsive moieties, thereby improving local drug accumulation and therapeutic persistence. However, despite their intrinsic advantages, the clinical performance of nanoparticle-based systems for scar intervention is highly dependent on penetration depth, cell-type specificity, release kinetics, biodegradability, and long-term safety.

In the context of fibroblast heterogeneity and phenotypic plasticity, nanoparticle-based systems are particularly suited for dynamic, microenvironment-adaptive targeting rather than static cell recognition. Ligand-functionalized nanoparticles, such as those targeting α-SMA, FAP, or ECM-associated receptors, preferentially accumulate in activated or pro-fibrotic fibroblast populations; however, the transient expression of these markers limits their universality across healing stages. To address this limitation, microenvironment-responsive nanoparticles that release payloads in response to elevated ROS, acidic pH, matrix metalloproteinase activity, or increased tissue stiffness can selectively engage pathological fibroblasts as they emerge during scar maturation, while sparing quiescent or reparative fibroblasts during early regeneration. This adaptability represents a key advantage of NDDSs in managing fibroblast phenotypic switching.

Although nanoparticles can traverse cellular membranes and access intracellular compartments, their penetration into the deep dermis—where pathological fibroblasts predominantly reside—remains a nontrivial challenge, particularly following topical administration. Passive diffusion alone is often insufficient, necessitating auxiliary strategies such as microneedles or bioadhesive formulations. Moreover, fibroblast heterogeneity and phenotypic plasticity further complicate selective targeting, raising concerns about off-target effects on keratinocytes, endothelial cells, or immune cells. As summarized in Table 1, diverse nanoparticle classes have demonstrated anti-scarring efficacy through distinct fibroblast-modulating mechanisms; however, their translational potential varies substantially depending on delivery context, safety margins, and long-term tissue interactions.

#### 5.1.1. Nanoparticles for Apoptosis and Ferroptosis Induction

Nanoparticle-mediated induction of apoptosis and ferroptosis represents a potent yet inherently aggressive anti-scarring strategy, primarily suited for conditions characterized by excessive fibroblast accumulation, such as HS. Cuprous oxide nanoparticles (CONPs) induce apoptosis in hypertrophic scar fibroblasts by triggering mitochondrial dysfunction, elevating intracellular ROS, and collapsing mitochondrial membrane potential [80]. While this mechanism efficiently reduces fibroblast density, its reliance on oxidative and mitochondrial stress raises concerns regarding collateral cytotoxicity, particularly in regenerating tissue where fibroblasts play essential reparative roles.

Similarly, supramolecular assemblies composed of cucurbit[7]uril (CB[7]), gold nanoclusters (AuNCs), and dihydroartemisinin (DHA) synergistically activate ferroptosis and apoptosis via lipid peroxidation and ROS amplification [83]. Although this dual-pathway approach achieved pronounced scar attenuation in rabbit ear models, the limited penetration depth and reliance on intradermal or microneedle-assisted delivery restrict its applicability to superficial or localized scars. More importantly, long-term safety remains insufficiently characterized, as sustained ferroptotic signaling may disrupt surrounding vasculature or delay epithelial regeneration. Thus, apoptosis/ferroptosis-inducing nanoparticles may be best positioned as short-term, localized interventions, rather than prolonged or prophylactic therapies.

#### 5.1.2. Nanoparticles for ROS Scavenging and Cytoprotection

In contrast to cytotoxic strategies, ROS-scavenging nanoparticles offer a more modulatory and biologically aligned approach to scar prevention by restoring redox homeostasis. Porous Se@SiO_2_ nanoparticles provide sustained selenium release and exhibit favorable biosafety profiles [84]. By attenuating ROS-induced fibroblast apoptosis and downregulating fibrotic markers (α-SMA, COL1A1, FN) while activating PI3K/Akt signaling, these nanoparticles support fibroblast survival and functional repair. Their topical applicability and low systemic toxicity enhance translational feasibility, although penetration into deeper fibrotic regions remains dependent on wound permeability.

**Figure 6:**
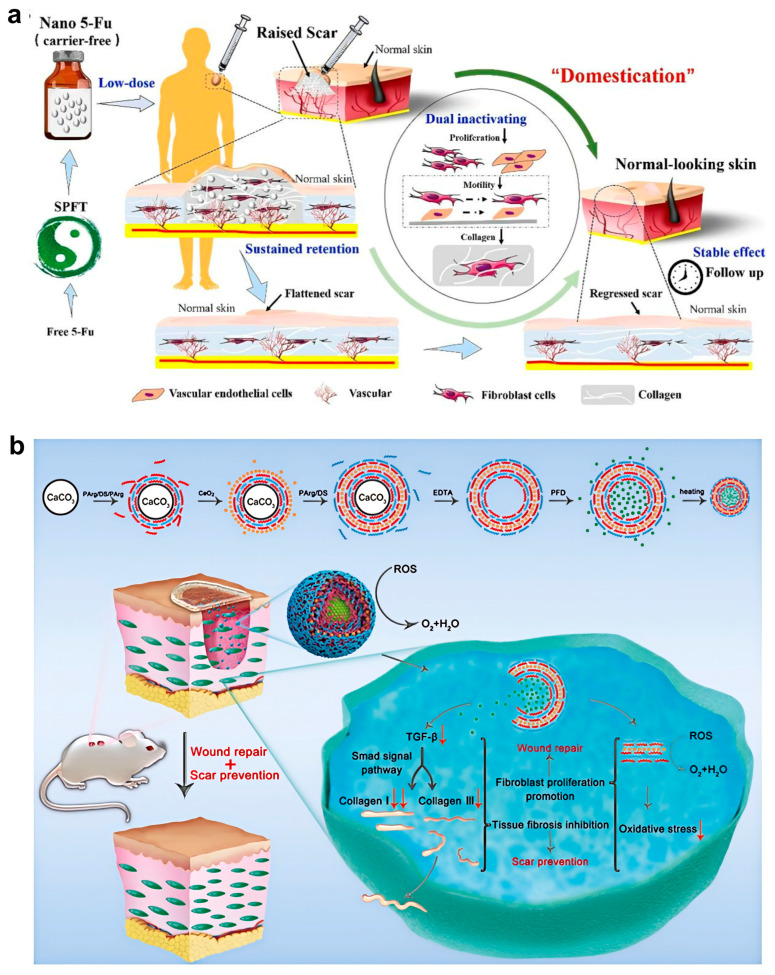
Application of nanoparticles in scar intervention. (**a**) Schematic illustration of the “domestication” process, in which low-dose intralesional nano 5-Fu exhibits sustained retention and simultaneously inactivates fibroblasts and vascular endothelial cells, thereby promoting scar regression and the restoration of normal-looking skin with durable long-term stability. Reprinted with permission from Ref. [89]. (**b**) Mechanistic illustration of CeO_2_ NPs in wound repair and scar prevention. The nanocomposites regulate ROS levels, suppress TGF-β/Smad signaling, and inhibit collagen overexpression, leading to reduced fibrosis and improved skin regeneration. Arrows in the figure represent the directional flow of ROS scavenging, TGF-β signaling modulation, and the effects on wound repair and scar prevention. Reprinted with permission from Ref. [85].

Cerium oxide nanoparticles (CeO_2_ NPs), through reversible Ce^3+^/Ce^4+^ redox cycling, mimic endogenous antioxidant enzymes, enabling continuous ROS buffering [85,94]. This catalytic behavior supports sustained regulation of oxidative stress, although it also raises concerns regarding biodegradability and nanoparticle persistence in tissue, necessitating careful optimization of dose and exposure duration. Consistent with this mechanistic paradigm, Figure 6b schematically illustrates how CeO_2_ nanozymes alleviate oxidative stress and downstream profibrotic signaling, thereby limiting collagen overproduction and favoring regenerative outcomes. Erbium borate nanoparticles (ErB-Nps) further exemplify a regenerative paradigm by inducing a fetal-like gene expression profile, promoting angiogenesis and ECM remodeling while suppressing fibrosis [86]. Collectively, ROS-scavenging nanoparticles demonstrate superior safety margins and broader therapeutic windows, making them attractive candidates for chronic wounds and early-stage scar prevention.

#### 5.1.3. Nanoparticles for Autophagy and Survival Pathways

Targeting autophagy represents a mechanistically nuanced strategy to restrain pathological fibroblast survival without overt cytotoxicity. Resveratrol-loaded mesoporous silica nanoparticles (MSN@Res) enhance intracellular delivery and stability of polyphenols, suppressing excessive autophagy via the ROS/p38-MAPK/HIF-1α/p53 axis [87]. While this approach effectively limits fibroblast viability and matrix production, its therapeutic outcome is highly sensitive to release kinetics, as prolonged autophagy inhibition may impair normal tissue remodeling. Furthermore, mesoporous silica frameworks require thorough biodegradation assessment, particularly in repetitive or long-term applications. Thus, autophagy-targeted nanoparticles require tight spatiotemporal control to avoid interference with physiological matrix remodeling.

#### 5.1.4. Microneedle-Enabled Delivery

Microneedle-assisted delivery effectively overcomes the stratum corneum barrier, thereby markedly enhancing intradermal deposition and local bioavailability of nanoparticle therapeutics. Carrier-free nano-5-Fu formulations exemplify this advantage, achieving prolonged retention, suppression of fibroblast and endothelial proliferation, and reduced collagen and microvessel density in rabbit models [89]. Beyond preclinical validation, a first-in-human randomized study demonstrated improved scar parameters with fewer adverse effects than free 5-Fu, highlighting the translational relevance of this strategy. As illustrated in Figure 6a, low-dose intralesional nano-5-Fu can achieve sustained local retention and concurrently suppress fibroblasts and vascular endothelial cells, thereby supporting durable scar regression. Nevertheless, microneedle systems introduce additional manufacturing complexity and regulatory considerations, particularly regarding mechanical reliability, sterility, and patient compliance. Their use may therefore be best suited for defined, accessible scars rather than widespread lesions.

### 5.2. Hydrogel–Nanocomposite Systems

Hydrogels are characterized by their high water content, cytocompatibility, tissue adhesion, and ECM-like mechanical properties, making them ideal candidates for wound management (Table 2 and Table 3) [95,96]. The hydration of these materials maintains a moist niche that prevents desiccation, alleviates pain, and supports re-epithelialization, significantly lowering the risk of scarring. Accumulating evidence indicates that hydrogel stiffness directly regulates fibroblast mechanosensing, thereby influencing cell migration, proliferation, and differentiation through force-dependent signaling pathways [97]. Stiff hydrogels, by enhancing the activation of FAK/Src signaling pathways, promote fibroblast adhesion and differentiation, facilitating the formation of myofibroblasts. However, prolonged exposure to high stiffness environments may lead to fibroblast dysfunction, causing pathological fibrosis. In contrast, softer hydrogels are often more effective in suppressing pathological fibrosis, especially when used in controlled drug-release systems [98].

These hydrogels offer a unique advantage in tissue engineering by combining rigidity with flexibility in a single system. Their viscoelastic properties play a critical role in modulating fibroblast behavior, including migration, differentiation, and mechanotransduction. By tuning the viscoelastic balance, these hydrogels can more closely mimic the dynamic mechanical environment of native tissues, providing fibroblasts with the necessary biomechanical cues for optimal healing, while avoiding excessive fibrosis. When combined with nanomedicines, “smart” hydrogels enable precise local drug delivery, staged release, and microenvironment-responsive behavior, which together regulate fibroblast migration, proliferation, and differentiation. This integration significantly enhances the translational potential of these systems for clinical applications, offering targeted and controlled therapeutic interventions in wound healing and scar prevention.

#### 5.2.1. Antibacterial and Pro-Regenerative Composites

A chitosan (CS)–hyaluronic acid (HA) composite hydrogel functionalized with gold nanoparticles (AuNPs) and FGFs has been developed to combine antibacterial activity with pro-regenerative signaling [99]. Mechanistically, CS provided antimicrobial adhesion, HA maintained hydration and ECM mimicry, AuNPs supported antibacterial/angiogenic effects, and FGF promoted fibroblast activation and neovascularization, accelerating wound closure and collagen maturation, particularly in diabetic wounds.

However, this composite material faces significant challenges in terms of penetration depth. For deep wounds or chronic wounds, local delivery effectiveness may be limited by the skin barrier, especially for materials that rely on passive diffusion. While CS and HA are known for their biodegradability, the rate of degradation could potentially mismatch the dynamic stages of wound healing, leading to either unstable drug release or premature degradation, which could compromise therapeutic outcomes. Thus, optimizing the mechanical properties and degradation characteristics of these hydrogels is essential for ensuring sustained drug delivery and tissue regeneration.

#### 5.2.2. Timed, Multi-Phase Modulation

Timed, multi-phase modulation has emerged as a rational strategy to align therapeutic interventions with the dynamic biological programs governing wound healing, enabling stage-specific regulation of inflammation, fibroblast activity, and matrix remodeling. For example, the liposome-encapsulated Methacryloyl gelatin (GelMA) hydrogel system co-delivering tetrahydrocurcumin (THC) and hepatocyte growth factor (HGF) exemplifies timed, multi-phase modulation for scarless wound healing [100]. In this system, early-stage antioxidant and anti-inflammatory effects mediated by THC prime the wound microenvironment, followed by HGF-driven fibroblast migration and collagen remodeling, and subsequent attenuation of TGF-β signaling to restrain fibrotic progression.

A central challenge of this strategy lies in achieving precise release kinetics, which requires not only temporal control but also coordinated synergy among multiple therapeutic agents. Temporal precision can also be achieved through pulsatile delivery strategies, such as photo-inducible imine-crosslinked hydrogels incorporating PLGA microcapsules for on-demand release of TGF-β inhibitors [101]. In murine, rabbit, and porcine models, this system enabled stage-specific interventions during the remodeling phase, leading to reduced fibroblast activation, collagen accumulation, and immune infiltration, while accelerating wound closure and limiting hypertrophic scarring. However, this approach requires extensive regulatory scrutiny and long-term clinical validation, especially in complex clinical settings.

#### 5.2.3. Peptide-Augmented Scaffolds

**Figure 7:**
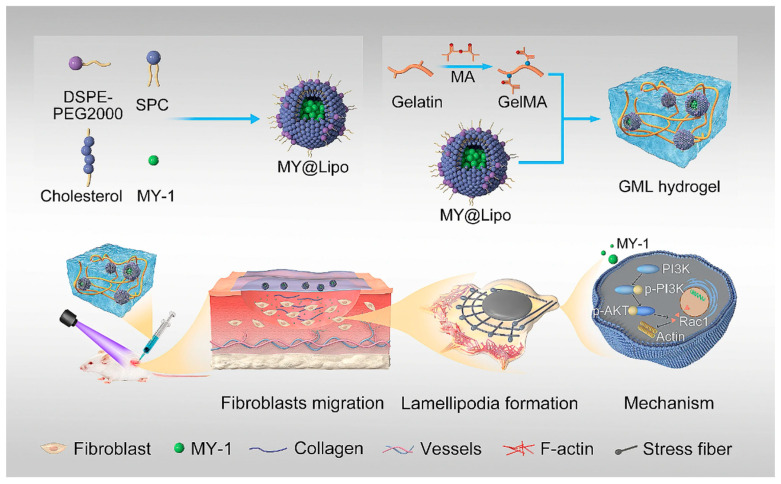
Fabrication and mechanism of GML for enhanced wound repair. The GML hydrogel promotes fibroblast migration and cytoskeletal remodeling via the PI3K/AKT/Rac1 signaling pathway, accelerating tissue regeneration and collagen remodeling. Reprinted with permission from Ref. [103].

Peptide-augmented scaffolds leverage bioactive peptides as instructive cues to directly reprogram wound repair processes. Peptide-augmented scaffolds exploit bioactive peptides as instructive cues to actively reprogram wound repair processes, shifting biomaterials from passive support toward dynamic regulation of the wound microenvironment. A self-assembling LA-peptide hydrogel attenuates excessive TGF-β signaling to coordinately regulate macrophage–fibroblast fate decisions and their pathological crosstalk, thereby suppressing myofibroblast activation and ECM overdeposition to enable scarless healing [102]. Meanwhile, a liposome-integrated MY-1 peptide GelMA hydrogel promotes fibroblast migration and tensile strength reinforcement through sustained activation of the PI3K/AKT–Rac1 axis, highlighting how peptide-functionalized matrices can precisely instruct cell motility and matrix remodeling during tissue regeneration [103]. As shown in Figure 7, the liposome-integrated GelMA platform encapsulates a representative design logic in which peptide-derived biochemical cues are converted into coordinated cytoskeletal remodeling and directional cell migration, ultimately supporting collagen remodeling and functional tissue restoration. Peptide-augmented scaffolds represent a paradigm shift in wound repair toward active microenvironmental instruction, wherein bioactive peptides orchestrate immune–fibroblast interactions, regulate cell migration, and restrain pathological matrix deposition, ultimately enabling scar-minimized and functionally restored tissue healing.

**Table 2:** In vivo nano-based hydrogel in skin scarring.

Nanomaterial	Biomolecule or Drug	Model	Administration Route	Stage	Major Outcomes	Ref.
Liposome-based multifunctional nanocomposite hydrogel	Tetrahydrocurcumin (THC) and hepatocyte growth factor (HGF)	Diabetic full-thickness skin wound model	Topical application	Preclinical	Improved wound healing and minimized scar formation via sustained THC and HGF release, with fibroblast and angiogenesis promotion.	[100]
Photo-inducible imine-crosslinked hydrogel with PLGA-NB microcapsules	TGF-β inhibitor	Murine, rabbit, and porcine skin wound healing models	Topical application	Preclinical	Enhanced scarless healing via TGF-β inhibitor release, with fibrosis reduction and accelerated tissue repair.	[101]
L-type anti-scar peptides hydrogel (LA-peptide hydrogel)	LA peptide	Mouse full-thickness skin wound model and rabbit ear HS model	Local application	Preclinical	Accelerated scarless wound healing via TGF-β attenuation–mediated regulation of macrophage–fibroblast crosstalk.	[102]
Herbal dual-network hydrogel (CZGF)	Chlorogenic acid (CA), bFGF and Zn^2+^	Staphylococcus aureus (MRSA) -infected diabetic mouse model and rabbit ear HS model	Topical application	Preclinical	Scar-free healing promotion: enhanced angiogenesis and reduced myofibroblast activation.	[104]
Polyvinyl alcohol (PVA) hydrogel	Hyperbranched polylysine (HBPL) and tannic acid (TA)	MRSA-infected rat full-thickness skin wound model and rabbit ear HS model	Topical application	Preclinical	Accelerated infected wound healing via antibacterial and ROS-scavenging effects, coupled with fibrosis attenuation and HS suppression.	[105]
Ag nanocomposite hydrogel (Ag NCH)	Ag nanoparticles and bFGF	MRSA-infected full-thickness rat skin wound mode	Topical application	Preclinical	Scar reduction via collagen remodeling and vascular maturation, promoting tissue regeneration.	[106]

**Table 3:** In vivo nano-based hydrogel in skin regeneration.

Nanomaterial	Biomolecule or Drug	Model	Administration Route	Stage	Major Outcomes	Ref.
Chitosan (CS)/HA hydrogel	Gold nanoparticles (AuNPs) and FGF	Streptozotocin-induced diabetic mouse full-thickness skin wound model	Topical application	Preclinical	Enhanced diabetic wound healing via AuNP-mediated antibacterial effects and FGF-promoted angiogenesis.	[99]
Methacryloyl gelatin (GelMA) hydrogel	MY-1 peptid	Rat full-thickness skin wound model	Topical application	Preclinical	Accelerated wound healing and enhanced tensile strength via MY-1 release, with promoted fibroblast migration and collagen synthesis.	[103]
Co-assembled supramolecular hydrogel (1 & SAB)	Salvianolic acid B (SAB) and phosphopeptide	Mouse full-thickness skin wound model	Topical application	Preclinical	Promoted wound repair through enhanced cell migration, angiogenesis, reduced oxidative stress, and suppressed fibrosis, with minimal scar formation.	[107]
Quaternary ammonium chitosan (QCS)/ tannic acid (TA) hydrogel	TA	Full-thickness skin wound model in rats, arterial and deep wounds (hemorrhagic models)	Injectable application	Preclinical	Rapid hemostasis with antibacterial and antioxidative effects, accelerating wound closure and tissue regeneration.	[108]
Collagen fibril–mimetic injectable nanofibrous hydrogel	Methylacrylyl hydroxypropyl chitosan (HM) and laponite (LAP)	Full-thickness skin wound model	Injectable application	Preclinical	Vascularization enhancement: promoted scarless healing and follicle neogenesis.	[109]

### 5.3. Liposome-Based Nanocarriers

Liposome-based nanocarriers have been extensively explored to enhance drug penetration, improve cellular uptake, and modulate fibroblast activity in both HS and chronic wounds (Table 4). Despite their broad application, the clinical utility of liposome-based systems remains strongly influenced by penetration depth, fibroblast specificity, release kinetics, biodegradability, and long-term safety profiles.

#### 5.3.1. Peptide-Functionalized Systems

Peptide-functionalized delivery systems have emerged as a promising approach for enhancing the transdermal delivery of therapeutic agents and modulating fibroblast behavior during wound healing and scar treatment. A representative example is TAT-modified liposomes encapsulating SAB, which significantly enhance cellular uptake and inhibit fibroblast proliferation and migration, while inducing apoptosis and downregulating TGF-β1 expression [110]. Similarly, dendritic lipopeptide-modified liposomes, designed to mimic viral transduction domains, enable dual penetration at both tissue and cellular levels, thereby improving intradermal drug accumulation and antifibrotic efficacy [111]. When loaded with triamcinolone acetonide, these liposomes disrupted fibroblast membranes, suppressed pro-inflammatory cytokines, remodeled collagen, and effectively reduced scarring in vivo. However, despite enhanced penetration, the specificity of peptide-functionalized liposomes toward pathological fibroblasts remains limited due to fibroblast heterogeneity and dynamic phenotypic switching during wound healing. As fibroblasts can change phenotypes during different stages of wound healing, targeting them with peptide-functionalized liposomes might not always guarantee efficacy, as fibroblast activation and marker expression can vary across individual wounds and healing phases.

Penetration depth remains a critical limitation, as liposomes largely rely on passive diffusion across the stratum corneum, restricting efficacy in deep dermal or fibrotic tissues. Even though TAT peptides can enhance cellular uptake, deeper tissue penetration remains hindered by the stratum corneum, limiting their effectiveness for deep wound or chronic tissue repair. Furthermore, peptide-functionalized systems require rigorous assessment of immune response and potential toxicity at high concentrations.

#### 5.3.2. Gene Delivery

**Figure 8:**
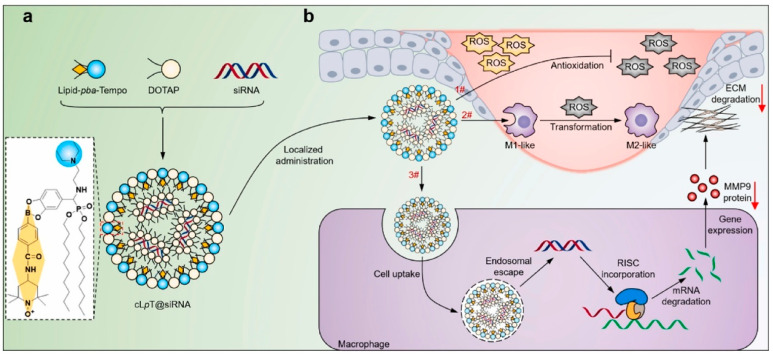
Schematic illustration of ROS-scavenging and siRNA-delivering lipid nanocomplexes (cLpT@siRNA) for diabetic wound healing. (**a**) The cLpT@siRNA nanocomplexes were prepared by assembling a cationic lipid, a ROS-responsive Tempo-conjugated lipid (Lipid-pba-Tempo), and MMP9-targeting siRNA (siMMP9) via electrostatic interaction. (**b**) Upon topical administration, the nanocomplexes co-deliver Tempo and siMMP9 to the diabetic wound site, restoring redox homeostasis, regulating macrophage polarization from M1-to M2-like phenotypes through ROS scavenging, and silencing MMP9 expression via RNA interference after endosomal escape—thereby promoting ECM remodeling and accelerating wound healing. The arrows indicate key biological processes, including cellular uptake, endosomal escape, and the implementation of RNA interference. Reprinted with permission from Ref. [113].

Recent advances in gene delivery systems have shown significant promise in enhancing wound healing, particularly in chronic wounds. A lipid-based nanocarrier (cLpT@siRNA) co-delivering MMP9-targeting siRNA and a ROS-scavenging lipid exemplifies this approach, enabling simultaneous modulation of oxidative stress and ECM remodeling [113]. By restoring redox balance, promoting macrophage polarization from M1 to M2 phenotypes, and enhancing neovascularization and collagen remodeling, this system effectively reshapes the wound microenvironment to accelerate wound closure. As schematically illustrated in Figure 8, the cLpT@siRNA nanocomplex integrates ROS scavenging with intracellular siRNA delivery to achieve coordinated regulation of macrophage phenotype and MMP9-mediated ECM remodeling in diabetic wounds. This example highlights how gene delivery systems can overcome the limitations of monotherapy by simultaneously targeting multiple pathological pathways, thereby synergistically enhancing tissue regeneration while mitigating fibrotic progression.

However, the challenge remains in ensuring the precise delivery of these dual therapeutic agents, as well as managing the long-term safety of the liposome carrier. The combined use of small molecules and genes within a single delivery vehicle necessitates comprehensive studies on their biodegradability, toxicity, and potential immune response over extended periods.

#### 5.3.3. Optimized Small-Molecule Delivery and PDT

Innovations in liposomal delivery systems have significantly improved the pharmacokinetics of small-molecule drugs and photodynamic therapy (PDT) in scar treatment. Liposomal 5-Fu enhanced intradermal retention and fibroblast uptake, outperforming free drug in suppressing proliferation, angiogenesis, and collagen deposition by downregulating TGF-β/Smad signaling in rabbit HS models [114]. Photodynamic therapy, when coupled with functional ethosomes co-loaded with 5-aminolevulinic acid (ALA) and catalase-mimicking nanocatalysts, demonstrated enhanced dermal penetration, hypoxia alleviation, and ROS generation under irradiation, reducing TGF-β expression and lowering scar elevation indices [115].

Despite these advantages, penetration depth remains a challenge. Liposomes and ethosomes are limited in their ability to penetrate deeper tissues and may require micro-needle-assisted delivery or other adjuvant technologies for effective deeper wound healing. Moreover, release kinetics of PDT systems require optimization to achieve timing precision for PDT efficacy, as poorly synchronized release of ALA or nanocatalysts may lead to inconsistent therapeutic effects. The biodegradability of liposomal formulations must also be considered, as liposomes can be prone to premature degradation, which might result in suboptimal drug release or off-target effects. Finally, the long-term safety of liposomal formulations must be evaluated in larger animal models and clinical settings, as the accumulation of liposomal carriers or the generation of ROS during PDT could lead to chronic inflammation, immune responses, or tissue damage if not properly managed.

### 5.4. Electrospun Nanofiber Materials

Electrospun nanofibers significantly influence cellular behavior through mechanotransduction pathways, though the specific mechanisms vary by cell type and fiber characteristics [116]. Aligned nanofibers promote myoblast polarization and myogenesis through Rac-related signaling pathways, while random fibers enhance RhoA/ROCK pathway activation, leading to increased stress fiber formation but reduced myogenic differentiation. In fibroblasts, low-density fibrous networks activate RhoA-ROCK signaling, promoting mechanotransduction and matrix remodeling capabilities through enhanced cytoskeletal assembly, cell contractility, and YAP nuclear translocation [117]. These electrospun nanofiber scaffolds can be functionally modified to provide topographical and biochemical cues that manipulate cell behaviors for skin scarring applications (Table 5) [118,119].

However, while these nanofiber-based materials offer significant advantages in wound healing and scar prevention, their clinical application is limited by several factors that must be carefully addressed for broader use in chronic wound management and long-term clinical interventions. These factors include penetration depth, fibroblast specificity, release kinetics, biodegradability, and long-term safety.

#### 5.4.1. Biomechanics-Tuned Dressings

Electrospun nanofibrous meshes, such as poly(3-hydroxybutyrate-co-3-hydroxyvalerate) (PHBV) and polyvinyl alcohol (PVA) combined with Pistacia atlantica (PAK) oleo-gum-resin, offer promising solutions for scar-free wound healing by optimizing biomechanical properties. The addition of 3-hydroxyvalerate (3HV) to PHBV enhances its elasticity, reducing excessive wound contraction and promoting smoother tissue regeneration [120]. In vivo, PHBV meshes with higher 3HV content reduced myofibroblast differentiation and supported the growth of skin appendages like hair follicles and sweat glands. Similarly, PVA/PAK nanofibrous membranes exhibited improved tensile strength and flexibility, significantly reducing wound contraction and promoting tissue regeneration in both excision and burn wound models [121]. These nanofiber-based dressings accelerated wound healing, increased collagen formation, and reduced inflammation, showcasing their potential as effective bioactive dressings. The combination of biomechanical optimization with bioactive effects positions these materials as promising candidates for enhancing wound healing outcomes.

However, biomechanical tuning through material composition and elasticity modulation may face challenges related to penetration depth when used for deep wounds or fibrotic tissues. Nanofiber meshes are often effective for superficial wounds, but their depth of penetration is limited, requiring complementary technologies like microneedles for enhanced delivery in deeper layers. Additionally, the release kinetics of bioactive agents embedded within the fibers must be precisely controlled to match the wound healing stages, as too rapid or delayed release may disrupt tissue regeneration and exacerbate fibrosis.

#### 5.4.2. Antioxidant and Responsive Systems

Electrospun PVP-Ce-Cur nanofibers combine ROS reduction, antimicrobial activity, and a microporous structure that promotes fibroblast proliferation, accelerating wound closure and limiting scarring [122]. A curcumin-loaded poly(octanediol citrate)/gelatin nanofibrous mat (CUR/POCA-Glt NM) was engineered to achieve scarless wound repair by exploiting MMP-2-responsive drug release [126]. The electrospun mat exhibited skin-like mechanical properties, strong wet adhesion, and rapid curcumin release in inflammatory microenvironments. In vivo studies demonstrated its efficacy across acute, chronic, and tension-loaded wounds, where it accelerated re-epithelialization, promoted collagen maturation, and mitigated hypertrophic scarring.

The release kinetics of curcumin, an antioxidant, play a crucial role in the efficacy of these materials. Curcumin has been shown to improve wound healing and reduce fibrosis, but its release rate must be optimally controlled to avoid excessive accumulation, which could lead to toxic effects or unregulated inflammation. The challenge lies in achieving sustained release while maintaining bioactivity, particularly in chronic wounds where the wound microenvironment is dysregulated.

#### 5.4.3. Multilayer Composites and Immune Modulation

**Figure 9:**
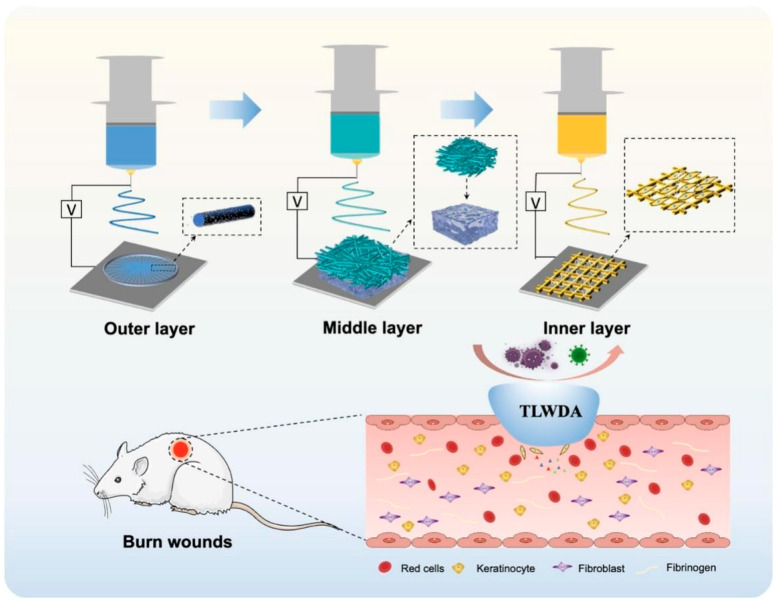
Application of electrospun nanofiber materials in scar intervention. Schematic diagram illustrating the fabrication of multi-layered nanofiber scaffolds for burn wound treatment. The process involves the sequential deposition of the outer, middle, and inner layers using electrospinning technology, each with distinct material properties. The scaffold is tested in an in vivo burn wound model, showcasing its interaction with red cells, keratinocytes, fibroblasts, and fibrinogen. The effects are associated with TLWDA. Reprinted with permission from Ref. [123].

Multilayer electrospun composites enable spatial segregation of biofunctions to coordinately regulate tissue regeneration and immune responses. As shown in Figure 9, a tri-laminate composite wound dressing (TLWDA) was engineered with (i) an outer aligned PCL nanofiber layer incorporating EGF to stimulate epidermal regeneration; (ii) a middle gelatin nanofiber-chitosan hydrogel composite providing structural integrity and antimicrobial action; and (iii) an inner mesh layer regulating adipose-derived stem cell (ADSC) paracrine signaling. This construct accelerated burn healing and improved angiogenesis, collagen organization, and epithelial regeneration [123]. Similarly, a QPSF/CeNPs nanofiber membrane accelerated burn healing, promoted tissue regeneration and collagen deposition through ROS modulation [124]. This membrane additionally stimulated endothelial cell proliferation and angiogenesis, modulated macrophage phenotypic polarization, and suppressed inflammatory responses.

The immune modulation effect of these multilayer composites is an important consideration, as chronic inflammation and immune dysregulation in wounds can impair healing and promote excessive scarring. Multilayer systems, while effective in enhancing cellular infiltration and promoting tissue regeneration, may also lead to immune overstimulation if not carefully optimized. Moreover, long-term safety and the biodegradability of the materials need to be addressed. For instance, PCL nanofibers are biocompatible, but their biodegradation rate must align with the wound healing process to avoid over-accumulation or long-term inflammatory responses.

## 6. Discussion

This review positions fibroblasts as central regulators of scar pathogenesis and critically evaluates NDDSs designed to modulate fibroblast activation, inflammatory signaling, and ECM remodeling. By explicitly linking delivery design parameters—including penetration enhancement, ligand-mediated targeting, and stimuli-responsive release—to fibroblast-centered mechanisms and functional outcomes, we establish a coherent framework for engineering anti-fibrotic interventions that attenuate pathological scarring while preserving the biomechanical and regenerative requirements of normal wound repair.

Despite encouraging preclinical progress, a major translational limitation is that many NDDSs claim “fibroblast targeting” without resolving which fibroblast states or subpopulations are actually engaged in vivo. Given the pronounced heterogeneity and temporal plasticity of fibroblasts during wound healing, indiscriminate suppression risks impairing essential reparative programs. Effective fibroblast-targeted NDDSs must therefore move beyond static, marker-based recognition and instead incorporate state-aware targeting strategies that leverage dynamic pathological cues—such as redox imbalance, matrix stiffening, aberrant protease activity, and pro-fibrotic cytokine gradients. Integrating ligand guidance with microenvironment-responsive release profiles offers a rational route to preferentially suppress pathological fibroblasts while sparing or even supporting reparative fibroblast functions during early regeneration.

At the mechanistic level, fibroblast behavior is governed by tightly coupled signaling networks rather than isolated pathways. Future studies should prioritize systematic interrogation of pathway coordination and temporal hierarchy to identify regulatory nodes that can be safely and durably modulated. In parallel, the influence of biomechanical forces and microenvironmental remodeling on fibroblast fate decisions remains underexplored and represents a critical axis for understanding—and potentially reversing—maladaptive scar formation.

From a translational perspective, substantial challenges remain in achieving precise spatiotemporal drug release, ensuring long-term biocompatibility, minimizing immunogenicity, and enabling scalable, reproducible manufacturing. Regulatory uncertainty further complicates clinical translation, as agencies such as the FDA and EMA have yet to establish comprehensive evaluation frameworks tailored to nanomedicines. Clarification is particularly needed regarding long-term toxicity, immunological consequences, and the complex pharmacokinetics of multifunctional nanomaterials.

Finally, while small-animal models have provided foundational insights, their limited ability to recapitulate human scar pathology—especially chronic inflammation and immune dysregulation—contributes to the persistent gap between preclinical efficacy and clinical outcomes. Future validation efforts should therefore incorporate more predictive models, including large-animal and humanized systems, alongside rigorously designed multicenter clinical studies.

In summary, fibroblasts represent a compelling and clinically relevant therapeutic target for scar modulation. Continued progress will depend on the interdisciplinary integration of materials science, regenerative biology, system-level mechanistic analysis, and emerging data-driven tools. By embracing fibroblast heterogeneity and disease-stage specificity as design constraints rather than obstacles, fibroblast-targeted NDDSs have the potential to advance scar therapy toward truly precise, stage-aware intervention and functional tissue regeneration.

## Figures and Tables

**Table 1:** Application of nanoparticles in skin scarring.

Nanomaterial	Biomolecule or Drug	Model	Administration Route	Stage	Major Outcomes	Ref.
Cuprous oxide nanoparticles (CONPs)	Cuprous oxide	Rabbit ear HS model	Intralesional injection	Preclinical	Scar attenuation and collagen deposition via mitochondrial dysfunction and ROS elevation.	[80]
Gold nanoclusters (AuNCs)	cucurbit[7]uril (CB[7]) and dihydroartemisinin (DHA)	Rabbit ear HS model	Microneedle patch	Preclinical	Scar attenuation via ferroptosis–apoptosis activation enabled by transdermal delivery.	[83]
Se@SiO_2_ nanoparticles	Selenium (Se)	Full-thickness excisional rat skin wound model	Topical application	Preclinical	Wound healing enhancement and scar attenuation via ROS modulation and PI3K/Akt pathway activation.	[84]
Cerium oxide nanoparticles (CeO_2_ NPs)	Pirfenidone (PFD)	Full-thickness rat skin wound model	Topical application	Preclinical	Accelerated wound healing and reduced scar formation via ROS scavenging–mediated TGF-β regulation, with enhanced fibroblast homeostasis and collagen remodeling.	[85]
Erbium borate nanoparticles (ErB-NPs)	Erbium borate	Full-thickness skin wound model	Topical application	Preclinical	Accelerated wound healing and scarless repair via oxidative stress reduction, angiogenesis promotion, and scarless gene-expression pattern modulation.	[86]
Resveratrol-laden mesoporous silica nanoparticles (MSN@Res)	Resveratrol (Res)	Human hypertrophic scar fibroblasts (HSFs) under hypoxic/ischemic conditions	In vitro administration	Preclinical	Scar attenuation via ROS-mediated p38-MAPK/HIF-1α/p53 modulation, with suppressed autophagy and enhanced apoptosis.	[87]
Bioadhesive nanoparticles (BNPs)	Verteporfin (VP)	Rat tail HS model	Intralesional injection	Preclinical	Scar attenuation via sustained YAP inhibition with reduced collagen deposition and angiogenesis.	[88]
Carrier-free pure 5-fluorouracil nanoparticles (nano 5-Fu)	5-fluorouracil (5-Fu)	Rabbit ear HS model	Intralesional injection	Clinical	Prolonged 5-Fu retention with reduced fibroblast proliferation, angiogenesis, and collagen deposition, leading to scar flattening and sustained efficacy with fewer injections.	[89]
PLGA nanoparticles	Asporin small interfering RNA (si-ASPN)	Nude mice keloid xenograft model	Intralesional injection	Preclinical	Keloid attenuation via ASPN silencing, with reduced fibroblast proliferation and collagen deposition.	[90]
Hyaluronic acid (HA)-modified zeolitic imidazolate framework-8 (ZIF-8) nano-vehicle	Curcumin	Infected third-degree burn mouse model	Topical application	Preclinical	Accelerated burn wound healing with pH-adaptive drug release, reduced inflammation and fibrosis, and enhanced antibacterial activity.	[91]
Human serum albumin nanoparticles (HSA NPs)	bFGF	Full-thickness rat skin wound model	Topical application	Preclinical	Accelerated wound healing via sustained bFGF delivery, with enhanced angiogenesis, fibroblast migration/proliferation, and reduced fibrotic differentiation.	[92]
Super carbonate apatite (sCA) nanoparticle	TIMP-1 siRNA (siTIMP-1)	Mouse HS model	Intralesional injection	Preclinical	Scar attenuation via TIMP-1 silencing–mediated collagen degradation.	[93]

**Table 4:** In vivo liposome-based nanocarriers in skin scarring.

Nanomaterial	Biomolecule or Drug	Model	Administration Route	Stage	Major Outcomes	Ref.
Cell penetrating peptide TAT-modified liposomes	SAB	Rat skin permeation	Topical application	Preclinical	Inhibited HSF proliferation, migration, and invasion, promoted apoptosis, reduced TGF-β1 expression, and enhanced cell penetration with sustained SAB release	[110]
Dual-penetrating arginine (R)-rich liposomal delivery platform (PRL)	Triamcinolone acetonide (TA)	Rabbit ear HS model	Topical application	Preclinical	Enhanced scar treatment via fibroblast apoptosis, collagen remodeling, and reduced fibroblast proliferation, with improved skin penetration and anti-inflammatory effects	[111]
PEG-liposomes	Simvastatin (SIM) and microRNA-21 plasmid (miR-21-P)	Full-thickness excisional wound model	Topical application	Preclinical	Synergistic wound healing via enhanced fibroblast migration, angiogenesis, and re-epithelialization, with reduced inflammation and improved tissue regeneration.	[112]
ROS-scavenging lipid nanoparticles	siRNA targeting MMP9	Diabetic wound model	Topical application	Preclinical	Accelerated wound healing via ROS scavenging, macrophage polarization, and siRNA delivery, enhancing neovascularization and collagen deposition.	[113]
5-Fu-loaded liposome (5-Fu-Lip)	5-Fu	Rabbit ear HS model	Intralesional injection	Preclinical	Improved scar treatment with reduced fibroblast activity, collagen deposition, and microvessel formation, while enhancing drug retention and minimizing side effects.	[114]

**Table 5:** In vivo electrospun nanofiber materials in skin scarring.

Nanomaterial	Biomolecule or Drug	Model	Administration Route	Stage	Major Outcomes	Ref.
Electrospun nanofibrous PHBV meshes	Poly(3-hydroxybutyrate-co-3-hydroxyvalerate) (PHBV)	Full-thickness wound model	Topical application	Preclinical	Reduced scar formation through mechanical modulation, decreased myofibroblast differentiation, and enhanced re-epithelialization and collagen organization.	[120]
PVA/pistacia atlantica (PAK) gum nanofibers	PAK	Rat excision and burn wound models	Topical application	Preclinical	Facilitated scarless wound healing through antioxidant effects, collagen remodeling, and improved skin regeneration, with increased GSH, catalase levels, and enhanced fibroblast activity.	[121]
Electrospun PVP-Ce-Cur nanofibers	Curcumin, cerium nitrate (Ce)	Full-thickness wound model	Topical application	Preclinical	Accelerated healing with re-epithelialization, minimal scarring, enhanced collagen deposition, and improved antioxidant activity.	[122]
Multifunctional electrospun nanofiber-based dressing	Adipose-derived stem cells (ADSCs)	Burn wound model	Topical application	Preclinical	Synergistic enhancement of burn wound healing through topographical, antibacterial, and ADSC-mediated regenerative regulation.	[123]
Silk fibroin-poly(e-caprolactone) polymer (PSF) electrospun	Cerium oxide nanoparticles (CeNPs)	Burn wound model	Topical application	Preclinical	Accelerated burn wound healing via ROS scavenging, enhanced angiogenesis, and M1 to M2 macrophage polarization, resulting in reduced inflammation, improved collagen deposition, and minimized scarring.	[124]
Polyurethane (PU) and HA-based electrospun nanofibers	20(R)-Ginsenoside Rg3	Rat third-degree burn model	Topical application	Preclinical	Enhanced burn wound healing with improved re-epithelialization, angiogenesis, and collagen deposition, and reduced inflammation and scarring, from PU/HA nanofibers and Rg3.	[125]
Poly[octanediol-co-(citric acid)] (POCA)-gelatin nanofibrous mat	Curcumin	Rat acute wound and diabetic chronic wound models	Topical application	Preclinical	Enhanced wound healing with accelerated collagen deposition, increased fibroblast activity, reduced oxidative stress, and minimal scarring in excision and burn models.	[126]
Cashew gum-polyvinyl alcohol (CGP-PVA) nanofibers	Cashew gum polysaccharide (CGP)	Rat excision and burn wound models	Topical application	Preclinical	Induced complete re-epithelialization with follicle regeneration and dense collagen matrix formation.	[127]
silk fibroin (SF)/PHBV nanofibers	Berberine (BBR)	Diabetic mouse wound and rabbit ear HS models	Topical application	Preclinical	Accelerated diabetic wound healing with improved angiogenesis, reduced scarring, and enhanced tissue regeneration through TGF-β1/Smad3 inhibition, and reduced inflammation.	[128]

## Data Availability

No new data were created or analyzed in this study.
